# Health Design Thinking: An Innovative Approach in Public Health to Defining Problems and Finding Solutions

**DOI:** 10.3389/fpubh.2020.00459

**Published:** 2020-08-28

**Authors:** Sylvie Abookire, Colin Plover, Rosemary Frasso, Bon Ku

**Affiliations:** ^1^Jefferson College of Population Health, Thomas Jefferson University, Philadelphia, PA, United States; ^2^Sidney Kimmel Medical College, Thomas Jefferson University, Philadelphia, PA, United States

**Keywords:** design thinking, human-centered design, education, public health, innovation

## Abstract

Current trends in the United States health care landscape call for innovative and adaptive approaches to improve outcomes and reduce inefficiencies. Design Thinking is an innovative approach to problem-solving that leverages insights from the end-users of new products, services, and experiences in order to develop best-fit solutions that are rapidly prototyped and iteratively refined. When compared to traditional problem-solving methods in health care and other public health adjacent fields, Design Thinking leads to more successful and sustainable interventions. Design Thinking has facilitated improvements in patient, provider, and community satisfaction, and in public health, has increased efficiency and collaboration in intervention development. Given the promising nature of Design Thinking as an effective problem-solving method, it follows that Design Thinking training would prove a beneficial addition to public health education. The integration of Design Thinking in public health education may equip public health leaders with essential skills necessary to understand and more effectively approach historically intractable challenges. This article describes the development and evaluation of a hands-on Design Thinking workshop, piloted with Master of Public Health (MPH) students in April, 2019 at Thomas Jefferson University. Preceding and following the workshop, evaluation forms were used to assess participants' knowledge about Design Thinking concepts and attitudes towards the workshop experience. Metrics were aligned with established learning objectives related to process, impact, and outcomes of the workshop. We hypothesized that the workshop intervention would increase participants understanding of Design Thinking concepts and applications in public health. Evaluations demonstrated that after attending the workshop, participants were able to understand and apply Design Thinking concepts in a public health context. Following the evaluation of pilot data, the workshop was refined and embedded in the MPH curriculum at Thomas Jefferson University in Philadelphia, PA.

## Introduction

Despite rapid advancements in research and technology, the United States health care system continues to endure pervasive inefficiencies including inequitable access, inconsistent quality, and high costs relative to comparable nations (1). Evidence suggests that innovation is an essential competency among the health care workforce to increase productivity and address these inefficiencies (2). Innovation is uniquely challenging in public health, as problems tend to be complex, dynamic, and context-specific, and can at times arise quickly and unpredictably, raising the urgency for rapid and efficient responses (3). However, current educational models in health care and public health provide limited training in creative thinking and innovation skills (2, 4). Thus, traditional public health education may be augmented through the inclusion of innovative, non-linear, adaptive, and cost-effective tools (5–8).

Design Thinking is one such tool; it is an innovative approach to problem solving that leverages insights from the end-users of new products, services, and experiences in order to develop best-fit solutions that are rapidly prototyped and iteratively refined so they can be deployed quickly and cost-effectively. It is a “human-centric” approach that involves collaboratively generating solutions alongside intended audiences, empathizing, developing a clear and succinct problem definition, creative ideation, and low-fidelity prototyping (9, 10). Design Thinking guides the early phases of innovation through deep empathy for users and a clear understanding of the problems facing them (11).

Frequently applied in industries such as business and technology, a Design Thinking approach recognizes that only an approximate 10% of new products or services successfully identify and respond to end-users' needs, meaning that the other 90% result in wasted time, funding, and other resources (11). In health care, Design Thinking has facilitated improvements in patient, provider, and community satisfaction, and can increase the efficiency and collaborative nature of intervention development (9, 12). When compared to traditional problem-solving methods in health care and public health, Design Thinking has demonstrated greater empathy for the needs of a community, a clearer understanding of the problem, more resource-efficient and cost-effective processes, and solutions with greater end-user satisfaction (12–15). For example, this approach has translated to increased use of public park spaces and improved efficacy of app-based behavior change interventions (12, 15). Furthermore, Design Thinking is well-suited to problem solving in the built environment, serving as a tool to combat health inequities and issues rooted in social determinants of health (9, 16). In partnership with the Center for Social Design at the Maryland Institute College of Art (MICA), the Baltimore City Health Department applied Design Thinking methods to support families with smoking cessation. They were trained in Design Thinking methods and interviewed families to understand barriers and gain empathy, which enabled them to develop an interactive pop-up event providing interventions informed by the needs of the target community and health behavior change theory, and supporting families in creating smoke-free zones in their homes (9). In another collaboration with MICA's Center for Social Design, Johns Hopkins Children's Center tackled pediatric asthma, which affects twice as many Baltimore children as the national average and disproportionately impacts black children. Their process involved observations and ethnographic research, followed by open-ended interviews with adolescent asthma patients, along with caregivers, advocates, and providers. Their ideation resulted in over 200 potential solutions (9).

Given the promising nature of Design Thinking applications in public health, it follows that Design Thinking training would prove a beneficial addition to public health curricula. Design Thinking fits neatly into formal public health education, which already emphasizes communication, teamwork, and qualitative approaches such as Community-Based Participatory Research (CBPR) (17). Design Thinking is well aligned with competencies outlined by the Council on Education for Public Health (CEPH), can be used to complement traditional public health methods, and prepares students to apply innovative and creative problem-solving methods to address challenges related to quality, cost, and access (18, 19).

This article describes the development and evaluation of a hands-on Design Thinking workshop, piloted with Master of Public Health (MPH) students at Thomas Jefferson University. This cross-disciplinary initiative aimed to provide Design Thinking training to public health students, preparing them to tackle complex problems efficiently and effectively as future professionals and providers. It challenged participants to engage directly with and apply material to public health problems. The goal of the endeavor was to integrate Design Thinking training as a core component of public health education in order to inspire widespread use of more systematic and effective approaches, and equip future leaders with innovative tools to improve the health of individuals and communities. The workshop provided an initial exposure to Design Thinking tools and applications, and aimed to increase participants' knowledge about Design Thinking while inspiring further curiosity about the pursuit of innovative methodologies in public health.

## Design and Development

The workshop was developed through a collaboration between the Health Design Lab and Colleges of Medicine and Population Health at Thomas Jefferson University to supplement traditional public health education. It was created by drawing upon the curriculum used in the Health Design Lab at Thomas Jefferson University and adapting the content for a public health audience. The participant worksheets and substantial content in the slide deck were adapted from the Design Thinking “Crash Course” and other materials openly accessible online from the Hasso Plattner Institute of Design at Stanford University (10). Both the Health Design Lab and Hasso Plattner Institute of Design granted permission for their content to be used.

**Figure 1:**
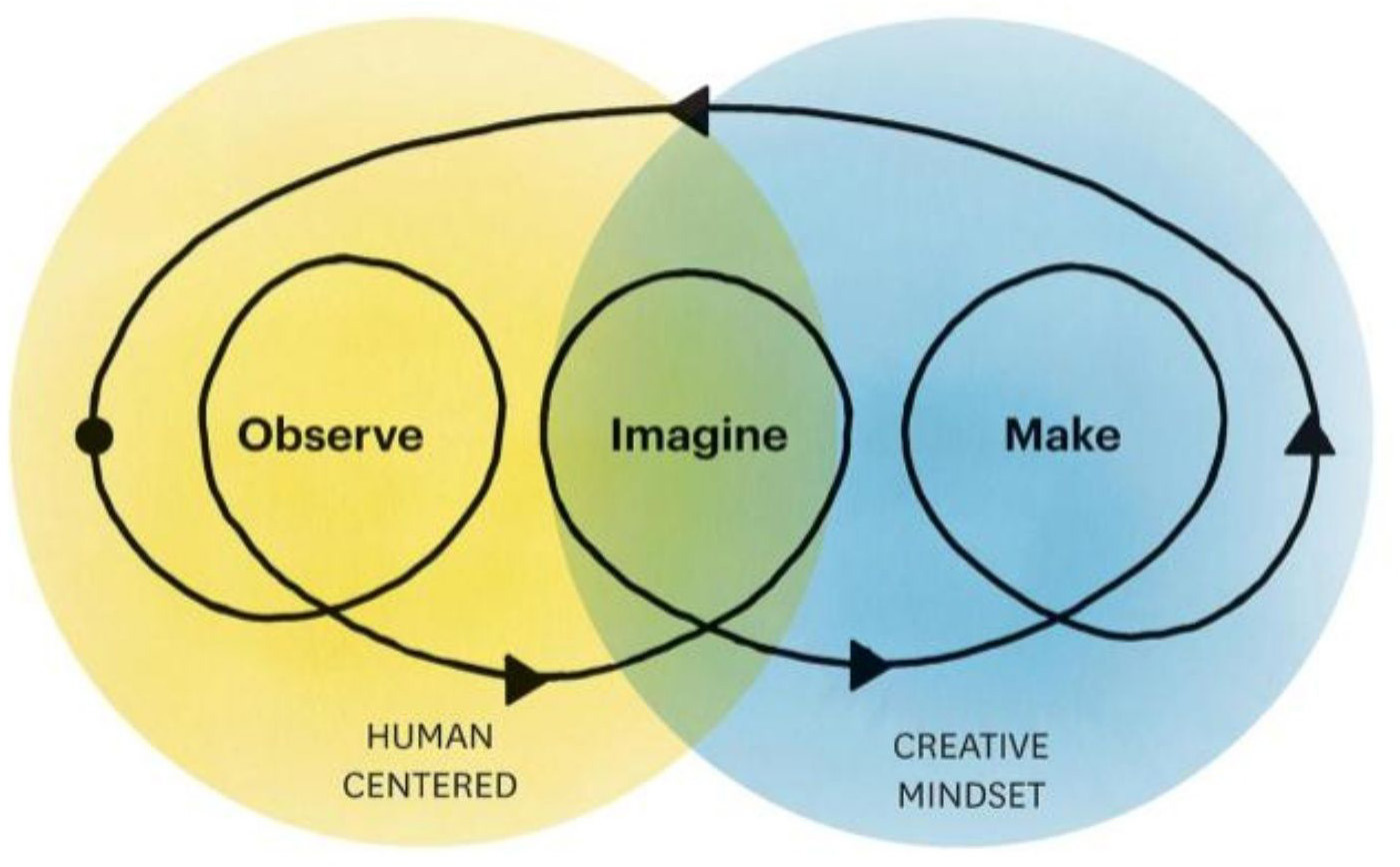
Fundamental principles and conceptual structure from Health Design Thinking (9).

The evidence-based workshop aims to teach Design Thinking within the context of public health. Principles from *Health Design Thinking* (Figure 1) and Stanford University's Hasso Plattner Institute of Design were used as conceptual structures (9, 10). The workshop occurs in person over the course of approximately two hours. It includes a brief, didactic introduction to Design Thinking methodology and reviews case studies demonstrating real-world applications of Design Thinking in public health, such as improving public park spaces and addressing social determinants of infant mortality inequities (12, 20). The remainder of the session involves hands-on activities centered on a design challenge related to health behavior change. Participants are paired into groups of two and guided through each of five stages in the Design Thinking process, beginning with interviewing one another to gain empathy into the other's barriers and facilitators to change, and concluding with prototyping and testing their innovative ideas. The learning objectives include several key concepts of Design Thinking, identified in Table 1. See Supplementary Materials for workshop agenda.

**Table 1:** Key concepts included in Design Thinking workshop (9, 10).

**Concept**	**Explanation**
**Human-centered**	Design Thinking is human-centered, meaning it is predicated on empathy for real users, understanding their needs, and developing a problem statement that matches these findings.
**Creative agency**	“Individuals' capacity to effect change in themselves and their situations to support successful creative problem-solving” (21).
**Empathy**	Understanding the user perspective is essential in developing solutions that solve the right problem and meet the needs of persons and populations impacted. Empathy can be gained through interviewing, observation, simulations, and co-design.
**Co-Design**	Co-design is the principle of designing alongside users from the beginning to end of the problem-solving process.
**Low fidelity prototyping**	Prototypes are generated quickly and with low fidelity materials such as markers, paper, cardboard, etc. This allows the designers to gather feedback without significant investment of time and resources.
**Fail fast**	The emphasis on low fidelity prototyping and solicitation of early and frequent user input is in part due to the benefits of a “fail fast” methodology, whereby time and resources are saved by avoiding expenditures on would-be failed solutions.
**Iterative design**	The Design Thinking process is iterative, meaning that steps are often returned to and repeated many times before a final solution is created.
**Bias toward action**	The Design Thinking process aims to promote action over individual thinking. Action could include discussion, questioning, soliciting feedback, drawing, creating, or prototyping.

## Implementation

### Setting

The workshop was piloted and evaluated on April 19, 2019 at Thomas Jefferson University from 12 p.m. to 3 p.m. with current public health graduate students. The pilot took place in the Health Design Lab space at Thomas Jefferson University. The lab space has several rectangular tables, each of which accommodated two individuals, therefore giving each participant ample space to sit and work creatively. Additionally, the Health Design Space offered use of their “prototyping cart,” which contained ample crafts supplies and facilitated low-fidelity prototyping.

### Participants

Ten current Thomas Jefferson University Master of Public Health (MPH) students participated in the workshop pilot. Participants were recruited from all “tracks” to MPH completion at Jefferson, including an accelerated one-year track, a part-time track, a full-time track, and a dual degree (MD/MPH) track. All students pursuing an MPH who expressed interested were included. A small sample size (ten participants) was chosen because experts suggest small groups are best for an effective Design Thinking workshop (9). MPH students were recruited via email, newsletter, messenger application, and word of mouth. They were compensated with lunch and clerkship credit hours that counted towards their degree requirements. In addition, several faculty members were present during the pilot to support, observe, and offer feedback for future iterations of the workshop.

### Evaluation Methods

Evaluations were collected using a paper survey that participants completed immediately before and after the workshop in order to assess their knowledge about Design Thinking and attitudes about the workshop experience. All survey data were anonymous and no identifying information was collected. The attitudes measured included interactivity, engagement, enjoyment, and learning. Interactivity, engagement and enjoyment were operationalized by asking participants to self-report their opinions of the workshop across each dimension using a Likert scale. For example, enjoyment was measured using the survey item: “Please rank your agreement with the following statement (1 = Strongly Disagree, 5 = Strongly Agree): I enjoyed the Design Thinking workshop.”

The attitudes items were only assessed after the workshop. Knowledge was assessed directly with a pre and post “quiz” style survey. Questions included: “Please describe three aspects of Design Thinking” and “What is one way that Design Thinking could be used in your career as a public health professional?” In addition, participants were asked to open-endedly report any comments or suggestions they had following the workshop. See Supplementary Materials for all survey questions. The workshop was ultimately evaluated for its adherence to several objectives related to process, impact, and outcomes, which built off of the survey items and are identified in Table 2.

**Table 2:** Workshop objectives.

**Objective**	**Objective Met (Yes/No)**
**PROCESS**	
On April 19, 2019 from 12 p.m. to 3 p.m. Design Thinking workshop will be piloted with 10 MPH students.	Yes
By July, 2019, an evidence-based Design Thinking workshop will be ready for use in PBH 501: Foundations of Public Health.	Yes
The workshop will take 2–3 h, and involve a combination of didactic learning, activity-based learning, and group discussion. Time will be allotted for breaks and unexpected challenges.	Yes
Participants will be told how much time is allotted for each activity.	Yes
Participants will be given opportunities ask questions, collaborate, make choices, and take breaks during workshop.	Yes
Workshop will take place in a space with tables and open space to promote collaboration and conversation.	Yes
**IMPACT**	
Immediately following workshop, participants who attend Design Thinking workshop will understand at least three Design Thinking concepts covered and appropriately identify at least one potential opportunity to use Design Thinking in their careers (assessed through survey).	Yes
Immediately following workshop, participants will report workshop was clear, enjoyable, and interactive (at least an average of 4/5 on a Likert scale survey).	Yes
**OUTCOME**	
By 2024, at least 10 Jefferson MPH graduates who attended workshop will report having used Design Thinking in their work to improve the health of individuals or communities.	TBD

### Curriculum Integration

Findings from the pilot were synthesized and the workshop delivery was refined accordingly prior to its integration into the curriculum at Thomas Jefferson University in the Fall of 2019. It is now offered as part of the Introduction to Public Health course, which is taken by all MPH students in the College of Population Health.

## Results

The survey evaluations generated both qualitative and quantitative findings. These data were analyzed, and despite the small sample size, proved to be valuable in refining the workshop prior to its integration into Jefferson's MPH curriculum.

### Quantitative Findings

Findings from the quantitative items on participant surveys are summarized here. The results indicated that participants' familiarity with Design Thinking and its applications increased meaningfully and that the workshop was received positively (Tables 3, 4).

**Table 3:** Participants' knowledge about Design Thinking.

**Question**	**Mean Before**	**Mean After**
“I am familiar with the concepts of Design Thinking and how to apply them.” (1 = Strongly Disagree, 5 = Strongly Agree)	2.3/5	4.4/5
“If you can, please describe three aspects of the Design Thinking process.” (Number of correct responses)	0.4/3	2.9/3
“What is one way you could incorporate Design Thinking into your work as a student, public health professional, or provider?” (Yes/no ability to answer question)	50% yes	100% yes

**Table 4:** Participants' attitudes about workshop experience.

**Question**	**Mean Response**
“I thought that the material in this workshop was presented clearly.” (1 = Strongly Disagree, 5 = Strongly Agree)	5/5
“I enjoyed this workshop.” (1 = Strongly Disagree, 5 = Strongly Agree)	5/5
“This workshop provided a good balance of hands on learning and lecture-style learning.” (1 = Strongly Disagree, 5 = Strongly Agree)	5/5

Overall, responses to the knowledge items indicate that participants were unfamiliar with Design Thinking prior to the workshop and were able to identify key concepts and potential applications for these concepts immediately following the workshop. On average, participants' self-reported familiarity with Design Thinking increased from 2.3 to 4.4 on a Likert Scale of 1–5. Number of correctly identified Design Thinking concepts increased from 0.4 to 2.9 on a scale from 0 to 3. An increase from 50 to 100% was observed among participants who were able to identify a meaningful public health application of Design Thinking relating to their own academic or professional careers.

Additionally, responses indicate positive attitudes towards the workshop experience. All workshop attendants indicated “5 (Strongly Agree)” to each question regarding workshop attitudes, indicating that the workshop was clear, enjoyable, and interactive.

### Qualitative Findings

Open-ended questions eliciting participants' comments and qualitative feedback indicated that the workshop was positively received. When asked what participants would change about the workshop, many said “nothing,” or “it was great.” Suggestions for improvements were limited. Participants were given approximately 10 minutes to create low-fidelity prototypes of their ideas. One participant requested additional time for prototyping a solution. Ideas prototyped included devices to assist with schedule management and mobile application interfaces to facilitate physical activity and dietary changes. Two participants indicated they would have liked an opportunity to share their prototypes. All other participants left spaces designated for constructive feedback and suggestions for improvements blank. Faculty feedback indicated that certain aspects of the directions and content needed clarification, including the roles of each partner during the design challenge, and the concept of “fail fast.” Following the pilot, all feedback was taken into consideration and workshop agenda, slides, and worksheets were refined accordingly.

## Discussion

This workshop module represents the first integration of Design Thinking training into the public health curriculum at Jefferson. The objective of the pilot was to demonstrate the feasibility and efficacy of a Design Thinking workshop in teaching MPH students key principles of Design Thinking. Our findings indicate that a workshop intervention can increase participants' knowledge of Design Thinking and its applications, and demonstrate the feasibility of integrating Design Thinking training into public health education. In addition, surveys revealed positive attitudes toward the workshop experience. This serves as a promising indication that students are likely to engage with and retain concepts, making them more likely to apply innovative approaches in public health. In order to build upon the success of this pilot and inspire widespread use of more innovative and empathy-driven approaches to improve the health of individuals and communities, this workshop was integrated into the core MPH curriculum at Jefferson.

There is additional potential to expand the content delivered in this workshop and further enhance its impact. The success of this pilot indicates that a full course on Design Thinking and other innovation tools, if developed within a public health program such as Jefferson's, might promote greater active learning and innovation among MPH students and future public health professionals. The Gillings School of Global Public Health at the University of North Carolina offers such a course, where students are taught creative prototyping, adopting an entrepreneurial mindset, and learning from failure (22, 23). Additionally, it is likely that the education provided through this workshop would benefit other health professional audiences, including public health students at other universities, public health practitioners, and educators.

Ultimately, the findings from this pilot should encourage other public health programs and educators to consider implementing a Design Thinking framework within public health education. Applying Design Thinking to public health challenges can help students, practitioners and educators to creatively and collaboratively problem solve. Integrating Design Thinking within public health pedagogy has the potential to increase use of creative approaches to develop more innovative ideas and interventions.

### Limitations

Although a small group size (10 participants in the pilot workshop) lent itself well to an interactive Design Thinking session, the amount of data collected was minimal and thus, statistical analyses could not be meaningfully performed. Additionally, the long-term impact of Design Thinking training on public health professionals' interventions and population level outcomes remains unclear. Finally, participants volunteered to participate in the session. It is possible that selection bias played a role in the responses to survey items, although we do not believe the findings to have been significantly influenced by bias.

### Future Research

Current and future offerings of this workshop within Jefferson's MPH program will continue to generate data using the same pre- and post-session evaluations that were used in the pilot. These data are currently used to refine the workshop, iterating such that future sessions adapt to the observations, feedback, and needs of participants. Additional data collection also provides the opportunity for a more robust statistical analysis, which will generate further evidence around the effectiveness of this intervention.

One of the goals of this pilot was to inspire more widespread use of Design Thinking in public health education and practice; as more Design Thinking trained professionals enter the public health workforce, opportunities to compare the effectiveness of intervention impact between those who did and did not receive Design Thinking training will become increasingly feasible. These comparisons will determine whether or not the integration of Design Thinking training into public health education truly serves to address the many intractable health and health care challenges faced nationally, and globally, today.

## Data Availability Statement

The raw data supporting the conclusions of this article will be made available by the authors, without undue reservation.

## Ethics Statement

Ethical review and approval was not required for the study on human participants in accordance with the local legislation and institutional requirements. Written informed consent for participation was not required for this study in accordance with the national legislation and the institutional requirements.

## Author Contributions

SA developed and implemented the workshop and accompanying materials and drafted the manuscript. BK contributed to content of workshop and generated the idea for the manuscript. CP contributed to development and refinement of the workshop and materials and including pre- and post-workshop evaluations. RF contributed to workshop refinement and edited the final version of the manuscript. CP and RF provided feedback and guided revision and iteration of workshop following pilot and facilitated curriculum integration process. All authors contributed to drafting and revisions, approved the final version for publication, and agreed to be accountable for the content of the work.

## Conflict of Interest

The authors declare that the research was conducted in the absence of any commercial or financial relationships that could be construed as a potential conflict of interest.

## Supplementary Material

Supplementary Table 1 Workshop agenda, concepts and activities.

Supplementary Table 2 Pre-workshop survey.

Supplementary Table 3 Post-workshop survey.

Supplementary Data Sheet 1 Participant worksheets.
