# Supplementary material for: Health Design Thinking: An Innovative Approach in Public Health to Defining Problems and Finding Solutions
Source: Front Public Health. 2020 Aug 28;8:459. doi: 10.3389/fpubh.2020.00459 (PMC7484480; doi:10.3389/fpubh.2020.00459)
Supplement: Supplementary Table 1 — Workshop agenda, concepts and activities. [file Table_1.DOCX]

| Time | Concept | Activity |
| --- | --- | --- |
| 12:00pm - 12:10pm | --- | People arriving, taking pre-surveys, lunch |
| 12:10pm - 12:20pm | Design thinking methodology, application in public health | Intro slides (didactic), lunch |
| 12:20pm - 12:45pm | Empathize  Open-ended questions  Co-design | Design challenge: interviews, debrief |
| 12:45pm - 12:55pm | Define the problem  Understanding user needs & insights | Synthesize insights, write POV/problem statements, debrief |
| 12:55pm - 1:10pm | Ideate  Empathize  Creative agency  Co-design | Generate solution sketches, share sketches with user and get feedback, debrief |
| 1:10pm - 1:15pm | Ideate | Revise solution based on feedback |
| 1:15pm -1:25pm | --- | BREAK  *Check out prototyping cart, begin thinking about how you’ll make this 3D* |
| 1:25pm-1:40pm | Bias towards action  Prototyping  Creative agency  Fail fast | Prototype solutions |
| 1:40pm - 1:55pm | Co-design  Fail fast  Empathize  Test | Testing, get feedback on prototypes, debrief, share prototypes |
| 1:55pm - 1:57pm | Iterative design | Iterative design (didactic) |
| 1:57pm - 2:02pm | Human-centered  Empathy  Creative agency  Prototype  Fail fast  Co-design  Bias towards action | Recap (didactic) |
| 2:02pm - 2:12pm | Empathy  Iterative design  Rapid prototyping  Applying design thinking in public health | Reflection questions |
| 2:12pm - 2:20pm | --- | Post-survey |
