# Supplementary material for: Health Design Thinking: An Innovative Approach in Public Health to Defining Problems and Finding Solutions
Source: Front Public Health. 2020 Aug 28;8:459. doi: 10.3389/fpubh.2020.00459 (PMC7484480; doi:10.3389/fpubh.2020.00459)
Supplement: Supplementary Table 2 — Pre-workshop survey. [file Table_2.DOCX]

Please answer all questions to the best of your ability. This is not to evaluate you, but to evaluate the program. Thank you!

1. **I am familiar with the concepts of design thinking and how to apply them.**

Strongly Disagree Disagree Unsure Agree Strongly Agree

1 2 3 4 5

1. **I have been exposed to design thinking previously.**

Strongly Disagree Disagree Unsure Agree Strongly Agree

1. 2 3 4 5
2. **If you can, please describe three aspects of the design thinking process.**
3. **What is one way you could incorporate design thinking into your work as a student, public health professional, or provider?**
