# Supplementary material for: Health Design Thinking: An Innovative Approach in Public Health to Defining Problems and Finding Solutions
Source: Front Public Health. 2020 Aug 28;8:459. doi: 10.3389/fpubh.2020.00459 (PMC7484480; doi:10.3389/fpubh.2020.00459)
Supplement: Supplementary Data Sheet 1 — Participant worksheets. [file Data_Sheet_1.PDF]

Your mission: **Redesign health behavior change** ...for your partner.

Jot down an interview guide 3min

d.

Start by gaining empathy.

1 Interview

8min (2 sessions x 4 minutes each)

Notes from your first interview

Switch roles & repeat Interview 1.

2 Dig deeper

8min (2 sessions x 4 minutes each)

Notes from your second interview

Switch roles & repeat Interview 2.

Reframe the problem.

3 Capture findings 3min

needs: things they are trying to do*

*use verbs

insights: new learnings about your partner's feelings/ worldview to leverage in your design*

*make inferences from what you heard

4 Define problem statement 3min

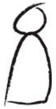

partner name/description

needs a way to

user's need

Surprisingly // because // but . . .

[circle one]

insight

Ideate: generate alternatives to test.

5 Sketch at least 5 *radical* ways to meet your user's needs. 5min

write your problem statement above

6 Share your solutions & capture feedback. 8min (2 sessions x 4 minutes each)

Notes

Switch roles & repeat sharing.

Iterate based on feedback.

7 Reflect & generate a new solution. 3min

Sketch your big idea, note details if necessary!

Build and test.

8 Build your solution.

Make something your partner can interact with!

[not here]

10min

9 Share your solution and get feedback.

✚ What worked...	▬ What could be improved...
? Questions...	! Ideas...

8min (2 sessions x 4 minutes each)
